# *trans*-Tetra­aqua­bis­[bis­(pyridin-3-yl)methanone-κ*N*]manganese(II) bis­(perchlorate)

**DOI:** 10.1107/S1600536811052184

**Published:** 2011-12-10

**Authors:** Bin Liu, Fan Zhang

**Affiliations:** a Department of Chemistry, Capital Normal University, Beijing 100048, People’s Republic of China

## Abstract

In the title complex, [Mn(C_11_H_8_N_2_O)_2_(H_2_O)_4_](ClO_4_)_2_, the Mn^2+^ ion is located on an inversion center with the slightly distorted N_2_O_4_ octa­hedral coordination sphere comprising N-atom donors from two monodentate *trans*-related bis­(pyridin-3-yl)methanone ligands and four water ligands. The two perchlorate anions are linked to the mononuclear complex mol­ecule through water O—H⋯O hydrogen bonds while inter-complex water O—H⋯N(pyridine) inter­actions form an infinite chain structure extending along the *b* axis. The perchlorate anions also function as inter-unit links through water O—H⋯O hydrogen bonds which, together with water O—H⋯O(carbon­yl) inter­actions, give a three-dimensional framework structure.

## Related literature

For background to coordination chemistry based on pyridyl­methanone derivatives, see: Huang *et al.* (2003 ▶); Chen *et al.* (2005 ▶); For transition metal complexes of bis­(pyridin-3-yl)methanone, see: Zhang (2011 ▶); Chen & Mak (2005 ▶).
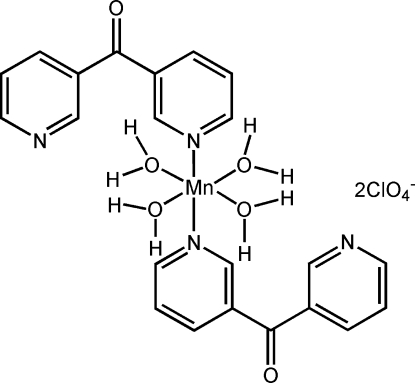


## Experimental

### 

#### Crystal data

[Mn(C_11_H_8_N_2_O)_2_(H_2_O)_4_](ClO_4_)_2_
*M* *_r_* = 694.29
Monoclinic, 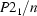

*a* = 8.410 (2) Å
*b* = 11.962 (3) Å
*c* = 14.386 (4) Å
β = 95.476 (5)°
*V* = 1440.6 (6) Å^3^
*Z* = 2
Mo *K*α radiation
μ = 0.72 mm^−1^
*T* = 296 K
0.45 × 0.32 × 0.25 mm

#### Data collection

Bruker APEXII CCD area-detector diffractometer
Absorption correction: multi-scan (*SADABS*; Bruker, 2007 ▶) *T*_min_ = 0.572, *T*_max_ = 1.000
7576 measured reflections
2529 independent reflections
1853 reflections with *I* > 2σ(*I*)
*R*_int_ = 0.076

#### Refinement

*R*[*F* ^2^ > 2σ(*F* ^2^)] = 0.050
*wR*(*F* ^2^) = 0.143
*S* = 1.04
2529 reflections
196 parameters
4 restraints
H-atom parameters constrained
Δρ_max_ = 0.48 e Å^−3^
Δρ_min_ = −0.51 e Å^−3^

### 

Data collection: *APEX2* (Bruker, 2007 ▶); cell refinement: *SAINT* (Bruker, 2007 ▶); data reduction: *SAINT*; program(s) used to solve structure: *SHELXS97* (Sheldrick, 2008 ▶); program(s) used to refine structure: *SHELXL97* (Sheldrick, 2008 ▶); molecular graphics: *SHELXTL* (Sheldrick, 2008 ▶); software used to prepare material for publication: *SHELXTL* and *PLATON* (Spek, 2009 ▶).

## Supplementary Material

Crystal structure: contains datablock(s) I, global. DOI: 10.1107/S1600536811052184/zs2167sup1.cif

Structure factors: contains datablock(s) I. DOI: 10.1107/S1600536811052184/zs2167Isup2.hkl

Additional supplementary materials:  crystallographic information; 3D view; checkCIF report

## Figures and Tables

**Table 1:** Hydrogen-bond geometry (Å, °)

*D*—H⋯*A*	*D*—H	H⋯*A*	*D*⋯*A*	*D*—H⋯*A*
O1*W*—H1*WA*⋯N1^i^	0.90	1.82	2.704 (4)	169
O2*W*—H2*WA*⋯O5^ii^	0.89	2.01	2.889 (5)	171
O2*W*—H2*WB*⋯O4	0.89	2.01	2.876 (4)	162
O1*W*—H1*WB*⋯O1^iii^	0.94	2.19	2.782 (3)	120

Symmetry codes: (i) 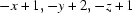; (ii) 
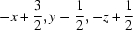
; (iii) 
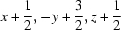
.

**Fig. 1.**
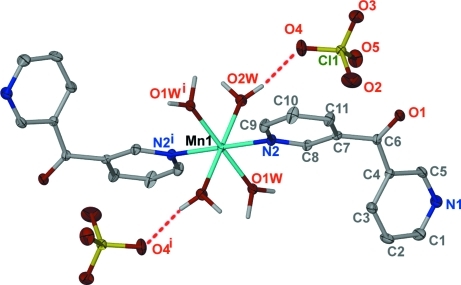
The title complex showing the atom-numbering scheme, with displacement ellipsoids shown at the 30% probability level. Hydrogen atoms are shown as spheres of arbitrary radius. Symmetry codes: (i) -x+1, -y+1, -z+1.

**Fig. 2.**
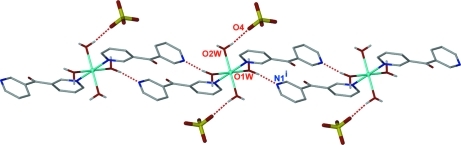
The infinite chain structure constructed through O—H···N interactions, extending along the b axial direction. All H atoms except those of the water molecules are omitted. Hydrogen bonds are shown as dashed lines.

## supplementary crystallographic information

### Comment

The coordination chemistry of the pyridyl ketone derivatives has attracted much attention due to the angular-shaped C(*sp*^2^)—CO—C(*sp*^2^) moiety and the rotatable C—C σ  bonds to the pyridyl groups (Huang *et al.*, 2003; Chen *et al.*, 2005). Recently, we reported the zigzag chain structure of the zinc(II) complex with bis(pyridin-3-yl)methanone (Zhang, 2011). Herein, we report the structure of a new mononuclear complex of this ligand with manganese(II), the title complex [Mn(C_11_H_12_N_2_O)_2_(H_2_O)_4_](ClO_4_)_2_.

In this complex, the Mn^2+^ ion is located on an inversion center with the slightly distorted N_2_O_4_ octahedral coordination sphere comprising N-atom donors from two monodentate *trans*-related bis(pyridine-3-yl)methanone ligands and four water ligands (Fig. 1). This is significantly different from that found in the polymeric complex [(CuL_2_)(ClO_4_)_2_]_n_ [L = bis(pyridin-3-yl)methanone], in which the N_4_O_2_ distorted octahedral Cu^2+^ center is surrounded by four equatorial pyridyl N-atom donors and two axially related perchlorate O-atoms (Chen *et al.*, 2005). In the present structure, the two perchlorate anions are linked to the mononuclear complex moiety through water O2*W*—H···O4 hydrogen bonds (Table 1) while inter-complex water O1*W*—H···N1(pyridyl) interactions form an infinite chain structure extending along the *b* axis (Fig. 2). The perchlorate anions also function as inter-unit links through water O2*W*—H···O4/O5 hydrogen bonds which with water O1*W*—H··· O1(carbonyl) interactions give a three-dimensional framework structure.

### Experimental

Bis(pyridin-3-yl)methanone was prepared according to the previously reported procedure (Chen & Mak 2005). The title complex was synthesized by reacting the ligand (19 mg, 0.1 mmol) with Mn(ClO_4_)_2_ . 6H_2_O (36 mg, 0.1 mmol) in 5 ml of methanol followed by the addition of 1 ml of deionized water. The clear solution obtained was stirred at room temperature for three hours, filtered and the filtrate left to slowly evaporate at room temperature. The block-like crystals were deposited after about three weeks (22.9 mg, 66% yield based on the ligand).

### Refinement

All H atoms were located in the difference electron density maps but were placed in idealized positions and allowed to ride on the carrier atoms, with C—H = 0.93 Å and with *U*_iso_(H) = 1.2*U*_eq_(C or O).

### Crystal data

**Table:**

[Mn(C_11_H_8_N_2_O)_2_(H_2_O)_4_](ClO_4_)_2_	*F*(000) = 710
*M**_r_* = 694.29	*D*_x_ = 1.601 Mg m^−^^3^
Monoclinic, *P*2_1_/*n*	Mo *K*α radiation, λ = 0.71073 Å
Hall symbol: -P 2yn	Cell parameters from 261 reflections
*a* = 8.410 (2) Å	θ = 2.2–27.2°
*b* = 11.962 (3) Å	µ = 0.72 mm^−^^1^
*c* = 14.386 (4) Å	*T* = 296 K
β = 95.476 (5)°	Block, colorless
*V* = 1440.6 (6) Å^3^	0.45 × 0.32 × 0.25 mm
*Z* = 2	

### Data collection

**Table:**

Bruker APEXII CCD area-detector diffractometer	2529 independent reflections
Radiation source: fine-focus sealed tube	1853 reflections with *I* > 2σ(*I*)
graphite	*R*_int_ = 0.076
ω scans	θ_max_ = 25.0°, θ_min_ = 2.2°
Absorption correction: multi-scan (*SADABS*; Bruker, 2007)	*h* = −10→9
*T*_min_ = 0.572, *T*_max_ = 1.000	*k* = −13→14
7576 measured reflections	*l* = −8→17

### Refinement

**Table:**

Refinement on *F*^2^	Primary atom site location: structure-invariant direct methods
Least-squares matrix: full	Secondary atom site location: difference Fourier map
*R*[*F*^2^ > 2σ(*F*^2^)] = 0.050	Hydrogen site location: inferred from neighbouring sites
*wR*(*F*^2^) = 0.143	H-atom parameters constrained
*S* = 1.04	*w* = 1/[σ^2^(*F*_o_^2^) + (0.0816*P*)^2^] *P* = (*F*_o_^2^ + 2*F*_c_^2^)/3
2529 reflections	(Δ/σ)_max_ < 0.001
196 parameters	Δρ_max_ = 0.48 e Å^−^^3^
4 restraints	Δρ_min_ = −0.51 e Å^−^^3^

### Special details

**Table:**

Geometry. All esds (except the esd in the dihedral angle between two l.s. planes) are estimated using the full covariance matrix. The cell esds are taken into account individually in the estimation of esds in distances, angles and torsion angles; correlations between esds in cell parameters are only used when they are defined by crystal symmetry. An approximate (isotropic) treatment of cell esds is used for estimating esds involving l.s. planes.
Refinement. Refinement of F^2^ against ALL reflections. The weighted R-factor wR and goodness of fit S are based on F^2^, conventional R-factors R are based on F, with F set to zero for negative F^2^. The threshold expression of F^2^ > 2sigma(F^2^) is used only for calculating R-factors(gt) etc. and is not relevant to the choice of reflections for refinement. R-factors based on F^2^ are statistically about twice as large as those based on F, and R- factors based on ALL data will be even larger.

### Fractional atomic coordinates and isotropic or equivalent isotropic displacement parameters (Å^2^)

**Table:**

	*x*	*y*	*z*	*U*_iso_*/*U*_eq_	
Mn1	0.5000	0.5000	0.5000	0.0230 (3)	
O1	0.1206 (3)	0.9254 (2)	0.26771 (19)	0.0445 (8)	
N1	0.3187 (5)	1.1514 (3)	0.4615 (3)	0.0486 (10)	
N2	0.2861 (3)	0.6001 (2)	0.4305 (2)	0.0298 (7)	
C1	0.3036 (6)	1.1271 (4)	0.5509 (3)	0.0560 (13)	
H1A	0.3305	1.1816	0.5958	0.067*	
C2	0.2504 (6)	1.0259 (4)	0.5794 (3)	0.0587 (14)	
H2A	0.2426	1.0122	0.6425	0.070*	
C3	0.2084 (5)	0.9447 (3)	0.5142 (3)	0.0443 (11)	
H3A	0.1721	0.8753	0.5322	0.053*	
C4	0.2211 (4)	0.9681 (3)	0.4213 (3)	0.0296 (9)	
C5	0.2765 (5)	1.0732 (3)	0.3991 (3)	0.0428 (11)	
H5A	0.2843	1.0895	0.3365	0.051*	
C6	0.1683 (4)	0.8891 (3)	0.3445 (3)	0.0304 (9)	
C7	0.1633 (4)	0.7661 (3)	0.3635 (2)	0.0272 (8)	
C8	0.2842 (4)	0.7106 (3)	0.4172 (3)	0.0300 (9)	
H8A	0.3684	0.7526	0.4455	0.036*	
C9	0.1591 (4)	0.5423 (3)	0.3913 (3)	0.0391 (10)	
H9A	0.1553	0.4656	0.4015	0.047*	
C10	0.0360 (5)	0.5911 (3)	0.3373 (3)	0.0500 (13)	
H10A	−0.0487	0.5478	0.3113	0.060*	
C11	0.0373 (5)	0.7041 (3)	0.3213 (3)	0.0403 (11)	
H11A	−0.0442	0.7382	0.2831	0.048*	
Cl1	0.58846 (12)	0.78266 (8)	0.19059 (7)	0.0363 (3)	
O2	0.4655 (4)	0.8174 (4)	0.2446 (3)	0.0884 (13)	
O3	0.5324 (4)	0.7861 (3)	0.0946 (2)	0.0706 (11)	
O4	0.6421 (5)	0.6736 (3)	0.2170 (2)	0.0752 (12)	
O5	0.7196 (4)	0.8584 (3)	0.2095 (3)	0.0671 (10)	
O1W	0.5725 (3)	0.6446 (2)	0.58278 (18)	0.0372 (7)	
O2W	0.6431 (3)	0.5430 (2)	0.38484 (19)	0.0429 (7)	
H1WA	0.6090	0.7146	0.5761	0.064*	
H2WA	0.6821	0.4815	0.3606	0.064*	
H2WB	0.6215	0.5865	0.3351	0.064*	
H1WB	0.5147	0.6440	0.6352	0.064*	

### Atomic displacement parameters (Å^2^)

**Table:**

	*U*^11^	*U*^22^	*U*^33^	*U*^12^	*U*^13^	*U*^23^
Mn1	0.0316 (5)	0.0164 (4)	0.0206 (4)	−0.0026 (3)	0.0008 (3)	0.0006 (3)
O1	0.069 (2)	0.0323 (15)	0.0293 (16)	0.0054 (14)	−0.0088 (14)	0.0023 (13)
N1	0.075 (3)	0.0299 (19)	0.041 (2)	−0.0167 (18)	0.0046 (19)	0.0002 (16)
N2	0.0303 (17)	0.0231 (17)	0.0355 (19)	−0.0009 (13)	0.0006 (14)	−0.0005 (14)
C1	0.087 (4)	0.037 (3)	0.043 (3)	−0.017 (2)	0.001 (3)	−0.010 (2)
C2	0.107 (4)	0.043 (3)	0.027 (2)	−0.024 (3)	0.008 (3)	−0.001 (2)
C3	0.071 (3)	0.029 (2)	0.033 (2)	−0.011 (2)	0.004 (2)	0.0025 (18)
C4	0.037 (2)	0.0231 (18)	0.029 (2)	−0.0018 (16)	0.0037 (17)	−0.0006 (16)
C5	0.066 (3)	0.033 (2)	0.029 (2)	−0.007 (2)	0.004 (2)	0.0059 (18)
C6	0.035 (2)	0.025 (2)	0.031 (2)	0.0033 (16)	−0.0004 (17)	0.0002 (16)
C7	0.029 (2)	0.027 (2)	0.026 (2)	0.0007 (15)	0.0037 (16)	−0.0037 (16)
C8	0.030 (2)	0.025 (2)	0.034 (2)	−0.0047 (15)	−0.0026 (17)	−0.0045 (16)
C9	0.037 (2)	0.025 (2)	0.055 (3)	−0.0073 (18)	0.004 (2)	−0.0016 (19)
C10	0.030 (2)	0.033 (2)	0.083 (4)	−0.0073 (18)	−0.013 (2)	−0.009 (2)
C11	0.032 (2)	0.034 (2)	0.053 (3)	0.0036 (18)	−0.008 (2)	−0.005 (2)
Cl1	0.0444 (6)	0.0336 (6)	0.0316 (6)	0.0038 (4)	0.0070 (4)	0.0028 (4)
O2	0.067 (3)	0.130 (4)	0.075 (3)	0.023 (2)	0.040 (2)	0.003 (3)
O3	0.097 (3)	0.073 (2)	0.039 (2)	0.025 (2)	−0.0151 (19)	−0.0047 (17)
O4	0.123 (3)	0.0360 (18)	0.064 (3)	0.012 (2)	−0.003 (2)	0.0135 (17)
O5	0.061 (2)	0.058 (2)	0.082 (3)	−0.0116 (17)	−0.0004 (19)	0.0085 (19)
O1W	0.0595 (18)	0.0210 (13)	0.0305 (15)	−0.0106 (12)	0.0009 (13)	−0.0003 (11)
O2W	0.0547 (18)	0.0373 (16)	0.0393 (17)	−0.0002 (14)	0.0173 (14)	0.0067 (13)

### Geometric parameters (Å, °)

**Table:**

Mn1—O1W^i^	2.155 (2)	C5—H5A	0.9300
Mn1—O1W	2.155 (2)	C6—C7	1.499 (5)
Mn1—O2W	2.199 (3)	C7—C11	1.386 (5)
Mn1—O2W^i^	2.199 (3)	C7—C8	1.386 (5)
Mn1—N2^i^	2.307 (3)	C8—H8A	0.9300
Mn1—N2	2.307 (3)	C9—C10	1.365 (6)
O1—C6	1.218 (4)	C9—H9A	0.9300
N1—C5	1.321 (5)	C10—C11	1.371 (6)
N1—C1	1.337 (6)	C10—H10A	0.9300
N2—C8	1.336 (4)	C11—H11A	0.9300
N2—C9	1.350 (4)	Cl1—O2	1.414 (4)
C1—C2	1.367 (6)	Cl1—O3	1.416 (3)
C1—H1A	0.9300	Cl1—O4	1.420 (3)
C2—C3	1.374 (6)	Cl1—O5	1.434 (3)
C2—H2A	0.9300	O1W—H1WA	0.9000
C3—C4	1.379 (5)	O1W—H1WB	0.9400
C3—H3A	0.9300	O2W—H2WA	0.8900
C4—C5	1.389 (5)	O2W—H2WB	0.8900
C4—C6	1.489 (5)		
			
O1W^i^—Mn1—O1W	180.00 (7)	N1—C5—H5A	118.0
O1W^i^—Mn1—O2W	85.27 (10)	C4—C5—H5A	118.0
O1W—Mn1—O2W	94.73 (10)	O1—C6—C4	119.8 (3)
O1W^i^—Mn1—O2W^i^	94.73 (10)	O1—C6—C7	120.1 (3)
O1W—Mn1—O2W^i^	85.27 (10)	C4—C6—C7	119.9 (3)
O2W—Mn1—O2W^i^	180.0	C11—C7—C8	118.6 (3)
O1W^i^—Mn1—N2^i^	89.47 (10)	C11—C7—C6	118.6 (3)
O1W—Mn1—N2^i^	90.53 (10)	C8—C7—C6	122.7 (3)
O2W—Mn1—N2^i^	89.29 (11)	N2—C8—C7	123.5 (3)
O2W^i^—Mn1—N2^i^	90.71 (11)	N2—C8—H8A	118.2
O1W^i^—Mn1—N2	90.53 (10)	C7—C8—H8A	118.2
O1W—Mn1—N2	89.47 (10)	N2—C9—C10	123.1 (4)
O2W—Mn1—N2	90.71 (11)	N2—C9—H9A	118.5
O2W^i^—Mn1—N2	89.29 (11)	C10—C9—H9A	118.5
N2^i^—Mn1—N2	180.000 (1)	C9—C10—C11	120.0 (4)
C5—N1—C1	117.2 (4)	C9—C10—H10A	120.0
C8—N2—C9	116.6 (3)	C11—C10—H10A	120.0
C8—N2—Mn1	125.0 (2)	C10—C11—C7	118.2 (4)
C9—N2—Mn1	117.9 (2)	C10—C11—H11A	120.9
N1—C1—C2	123.0 (4)	C7—C11—H11A	120.9
N1—C1—H1A	118.5	O2—Cl1—O3	109.5 (2)
C2—C1—H1A	118.5	O2—Cl1—O4	110.7 (3)
C1—C2—C3	119.4 (4)	O3—Cl1—O4	110.8 (2)
C1—C2—H2A	120.3	O2—Cl1—O5	107.5 (2)
C3—C2—H2A	120.3	O3—Cl1—O5	110.2 (2)
C2—C3—C4	118.7 (4)	O4—Cl1—O5	108.1 (2)
C2—C3—H3A	120.6	Mn1—O1W—H1WA	140.3
C4—C3—H3A	120.6	Mn1—O1W—H1WB	107.0
C3—C4—C5	117.7 (4)	H1WA—O1W—H1WB	108
C3—C4—C6	123.1 (3)	Mn1—O2W—H2WA	110.5
C5—C4—C6	119.1 (3)	Mn1—O2W—H2WB	131.2
N1—C5—C4	124.0 (4)	H2WA—O2W—H2WB	102.9
			
O1W^i^—Mn1—N2—C8	−150.3 (3)	C5—C4—C6—O1	−25.7 (6)
O1W—Mn1—N2—C8	29.7 (3)	C3—C4—C6—C7	−25.7 (6)
O2W—Mn1—N2—C8	−65.0 (3)	C5—C4—C6—C7	158.5 (4)
O2W^i^—Mn1—N2—C8	115.0 (3)	O1—C6—C7—C11	−35.3 (5)
O1W^i^—Mn1—N2—C9	22.3 (3)	C4—C6—C7—C11	140.5 (4)
O1W—Mn1—N2—C9	−157.7 (3)	O1—C6—C7—C8	141.0 (4)
O2W—Mn1—N2—C9	107.5 (3)	C4—C6—C7—C8	−43.3 (5)
O2W^i^—Mn1—N2—C9	−72.5 (3)	C9—N2—C8—C7	−2.4 (6)
C5—N1—C1—C2	1.4 (8)	Mn1—N2—C8—C7	170.3 (3)
N1—C1—C2—C3	−0.7 (9)	C11—C7—C8—N2	0.1 (6)
C1—C2—C3—C4	−0.1 (8)	C6—C7—C8—N2	−176.1 (4)
C2—C3—C4—C5	0.2 (7)	C8—N2—C9—C10	2.5 (6)
C2—C3—C4—C6	−175.6 (4)	Mn1—N2—C9—C10	−170.6 (4)
C1—N1—C5—C4	−1.3 (7)	N2—C9—C10—C11	−0.4 (7)
C3—C4—C5—N1	0.5 (7)	C9—C10—C11—C7	−1.9 (7)
C6—C4—C5—N1	176.5 (4)	C8—C7—C11—C10	2.0 (6)
C3—C4—C6—O1	150.1 (4)	C6—C7—C11—C10	178.4 (4)

Symmetry codes: (i) −*x*+1, −*y*+1, −*z*+1.

### Hydrogen-bond geometry (Å, °)

**Table:**

*D*—H···*A*	*D*—H	H···*A*	*D*···*A*	*D*—H···*A*
O1W—H1WA···N1^ii^	0.90	1.82	2.704 (4)	169
O2W—H2WA···O5^iii^	0.89	2.01	2.889 (5)	171
O2W—H2WB···O4	0.89	2.01	2.876 (4)	162
O1W—H1WB···O1^iv^	0.94	2.19	2.782 (3)	120

Symmetry codes: (ii) −*x*+1, −*y*+2, −*z*+1; (iii) −*x*+3/2, *y*−1/2, −*z*+1/2;  (iv) *x*+1/2, −*y*+3/2, *z*+1/2.
